# Postoperative Stroke after Spinal Anesthesia and Responses of Carotid or Cerebral Blood Flow and Baroreflex Functionality to Spinal Bupivacaine in Rats

**DOI:** 10.3390/biology10070617

**Published:** 2021-07-02

**Authors:** Yan-Yuen Poon, Yueh-Wei Liu, Ya-Hui Huang, Samuel H. H. Chan, Ching-Yi Tsai

**Affiliations:** 1 Department of Anesthesiology, Chang Gung Memorial Hospital, Kaohsiung 83301, Taiwan; elephant423@gmail.com; 2 Institute for Translational Research in Biomedicine, Chang Gung Memorial Hospital, Kaohsiung 83301, Taiwan; sukiclock@gmail.com; 3 Department of General Surgery, Chang Gung Memorial Hospital, Kaohsiung 83301, Taiwan; anthony0612@me.com

**Keywords:** spinal anesthesia, carotid and cerebral blood flow, baroreflex-mediated sympathetic vasomotor tone, hypotension, bradycardia, stenosis, atheromatous lesions

## Abstract

**Simple Summary:**

Spinal anesthesia (application of local anesthetic into the subarachnoid space of spinal cord where cerebrospinal fluid circulates) is generally considered a simple, effective and safe procedure. Three rare incidents of patients who developed stroke after surgery under spinal anesthesia with bupivacaine prompted us to report these infrequent clinical cases. Their shared pathology of stenosis (abnormal narrowing of a blood vessel) or atheromatosis (abnormal accumulation of material in an artery) in the carotid or middle cerebral artery (key blood supply to brain), revealed postoperatively, formed the impetus to assess in a companion animal study whether spinal bupivacaine may compromise blood supply to the brain. We found in two-thirds of the rats studied that on application of bupivacaine into the subarachnoid space, blood pressure, blood flow in common carotid artery and baroreflex (responsible for maintained blood pressure) remained stable after a transient drop. However, the other third exhibited a secondary hypotension, depressed baroreflex, declined heart rate, reduced carotid blood flow and waning blood supply to and oxygen level in the cerebral cortex. Our animal study confirmed that blood supply to the brain can indeed be compromised (cause of stroke) after spinal anesthesia, and an impaired baroreflex, which leads to hypotension, plays a contributory role.

**Abstract:**

Spinal anesthesia is generally accepted as an effective and safe practice. Three rare incidents of postoperative cerebral infarction after surgery under spinal anesthesia prompted us to assess whether spinal bupivacaine may compromise carotid or cerebral blood flow. Postoperative examination after the stroke incident revealed that all three patients shared a common pathology of stenosis or atheromatosis in the carotid or middle cerebral artery. In a companion study using 69 Sprague-Dawley rats, subarachnoid application of bupivacaine elicited an initial (Phase I) reduction in the mean arterial pressure, carotid blood flow (CBF) and baroreflex-mediated sympathetic vasomotor tone, all of which subsequently returned to baseline (Phase II). Whereas heart rate (HR) exhibited sustained reduction, cardiac vagal baroreflex, baroreflex efficiency index (BEI) and tissue perfusion and oxygen in the cerebral cortex remained unaltered. However, in one-third of the rats studied, Phase II gave way to Phase III characterized by secondary hypotension and depressed baroreflex-mediated sympathetic vasomotor tone, along with declined HR, sustained cardiac vagal baroreflex, decreased BEI, reduced CBF and waning tissue perfusion or oxygen in the cerebral cortex. We concluded that carotid and cerebral blood flow can indeed be compromised after spinal anesthesia, and an impaired baroreflex-mediated sympathetic vasomotor tone, which leads to hypotension, plays a contributory role.

## 1. Introduction

Spinal anesthesia has been a widely used anesthetic technique since it was introduced to the surgical world by August Bier in 1898 [1]. It is generally considered a simple procedure with a high success rate; anesthesiologists master spinal anesthesia after only 40 to 70 supervised attempts [2,3]. Recent reports [4,5,6] showed that spinal anesthesia has advantages over general anesthesia in short term morbidity and mortality among the elderly who undergo total knee or total hip replacement.

The most reported common side effects of spinal anesthesia are hypotension and bradycardia [7,8] or drowsiness [9,10], which are believed to be the cardiovascular consequences of induced preganglionic sympathetic paralysis [11,12]. Hypotension occurs from a sympathetic block that leads to decreases in systemic vascular resistance and central venous pressure [13,14,15,16]. Bradycardia occurs from a shift in the cardiac autonomic balance towards the parasympathetic system in high spinal anesthesia [17], or reverse Bainbridge reflex in low spinal anesthesia [18]. Intraoperative drowsiness may be related to spinal anesthesia-induced hypotension or arterial oxygen desaturation [6,19].

The baroreflex is the most fundamental feedback mechanism in the short-term [20] and long-term [21] regulation of blood pressure (BP) and heart rate (HR). Cerebral autoregulation is the intrinsic ability of the brain to maintain stable cerebral blood flow despite changing mean arterial pressure (MAP) [22]. Of the limited literature on the effects of spinal anesthesia on these two fundamental regulatory processes on cardiovascular functions, Gratadour and colleagues [23] reported no significant change in the spontaneous cardiac baroreflex during spinal anesthesia with bupivacaine in patients scheduled for elective inguinal hernia repair. Small but statistically significant reduction of cerebral blood flow has been shown during spinal anesthesia in the very elderly [24] and preterm infants [25]. Perioperative stroke is a devastating complication associated with high morbidity and mortality [26,27,28,29], and postoperative stroke is uncommon in noncardiac or nonneurologic surgery [30,31].

Three rare incidents of patients who developed cerebral infarction after surgery under spinal anesthesia with bupivacaine prompted us to report these infrequent clinical cases. Their shared common pathology of stenosis or atheromatous lesions in carotid or middle cerebral artery revealed on further examination after the incident formed the impetus for our companion animal study with a primary aim to assess the guiding hypothesis that intrathecal administration of bupivacaine may compromise carotid and cerebral blood flow, and baroreflex dysfunction may play a contributory role.

## 2. Materials and Methods

### 2.1. Ethics Statement

A retrospective report of the three clinical cases was approved by the Institutional Review Board of the Chang Gung Memorial Hospital (IRB approval number: 202000449B0C501). All methods were carried out in accordance with the Declaration of Helsinki and the HIPAA Privacy Rule. The Board also waived informed consent from the patients because they have been de-linked. All experimental procedures carried out on the laboratory animals were approved by the Institutional Animal Care and Use Committee of the Kaohsiung Chang Gung Memorial Hospital (IACUC approval number: 2019062001). The methods were performed in accordance with the ARRIVE guidelines and were in compliance with the Animal Protection Law set forth by the Council of Agriculture, Taiwan and the AAALAC—International Guide for the Care and Use of Laboratory Animals. All efforts were made to reduce the number of animals used and to minimize animal suffering during the experiment.

### 2.2. Patients

As indicated in the Introduction, the immediate impetus of this study arises from 3 incidents out of 198 patients who received surgery under spinal anesthesia performed by YYP between February 2014 and January 2018 developed cerebral infarction 4–8 h after discharge from the postanesthetic care unit (PACU). Both patient A, a 64-year-old woman, and Patient B, a 77-year-old man, suffered from knee osteoarthritis and were scheduled for total knee arthroplasty. Patient C, a 72-year-old man, suffered from left inguinal hernia and was scheduled for herniorrhaphy. Spinal anesthesia was performed with 3 mL of 0.5% bupivacaine injected through the L4-L5 interspace into the lumbar subarachnoid space. Patients were adequately pre-hydrated with crystalloid solution before spinal anesthesia. Pre-anesthesia MAP and HR of the three patients were: Patient A, 102 mmHg and 72 beats/min; Patient B, 115 mmHg and 68 beats/min; Patient C, 118 mmHg, 67 beats/min. MAP was transiently suppressed by 10–13% of the baseline values for several min on administration of bupivacaine, then gradually returned to pre-anesthesia levels. The HR of the three patients were persistently reduced by 5 to 10% of the pre-anesthesia values. Intraoperative course was smooth and patients were returned to the wards after one hour of observation in the PACU. The three patients were not prescribed bed rest after returning to the wards [32]. Besides postoperative cerebral infraction, these three patients suffered no other complications, including myocardial infarction, before they were discharged from the hospital.

### 2.3. Animals

**Figure 1:**
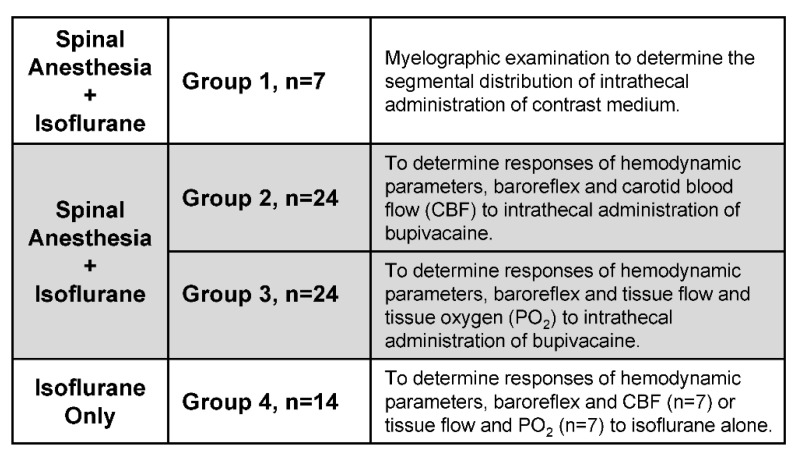
Experimental setup and animals used in each group in this study.

Experiments were carried out on specific pathogen-free adult male Sprague-Dawley rats (body weight: 300–350 g; age: 9–10 weeks; *n* = 69) purchased from BioLASCO (Taipei City, Taiwan), Taiwan. They were housed two per cage under a 12:12-h light-dark cycle and temperature control (24–25 °C) in an AAALAC International-accredited Center for Laboratory Animals. Standard laboratory rat chow and tap water were available ad libitum. Animals were randomly assigned to four groups (Figure 1) (see Section 3.2.1 for a detailed description).

### 2.4. Subarachnoid Catheterization

**Figure 2:**
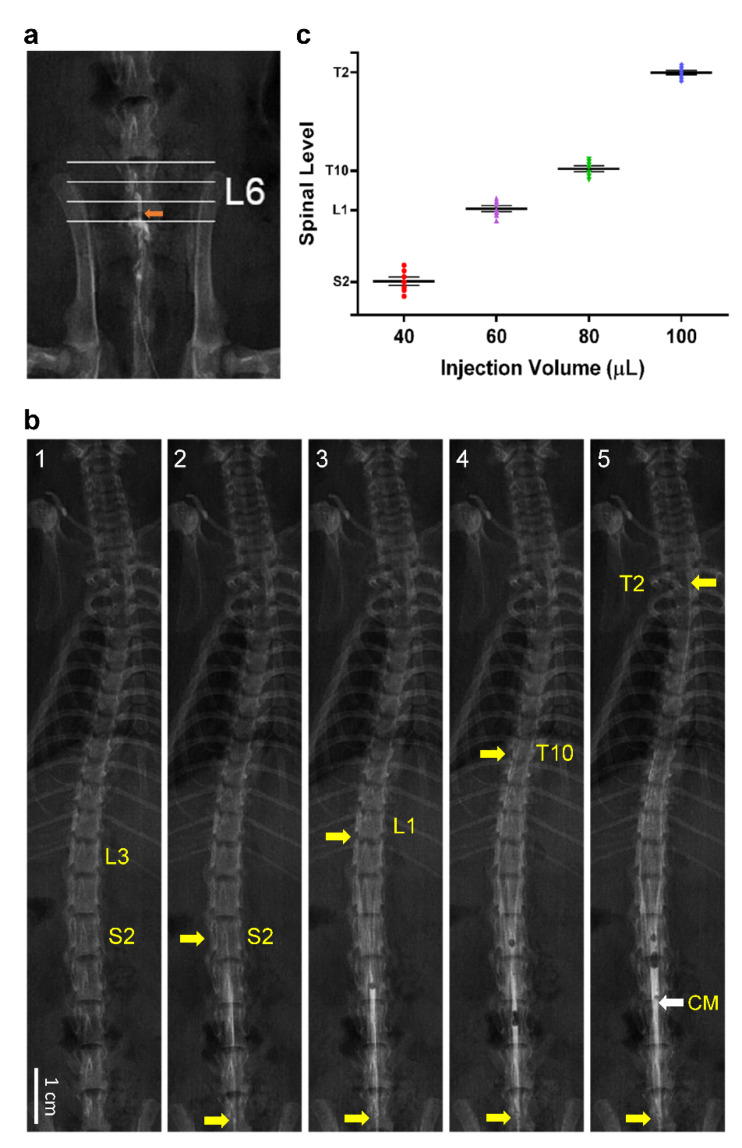
(**a**) Demonstration of lodging of the tip of the catheter (orange arrow) in the subarachnoid space below the middle portion of L6 vertebra. (**b**) Representative examples of myelographic examination before (1) and after intrathecal administration of four successive doses of contrast medium, given at 40 (2), 60 (3), 80 (4) or 100 (5) μL. Note that all demarcations denote levels of the spinal cord: the lower yellow arrows mark the location of the tip of the catheter, and the upper yellow arrows indicate the highest points reached by the enhanced roentgenological images. CM: conus medullaris. (**c**) Scattered plots showing the extent of dispersion in the spinal cord of contrast medium on intrathecal administration at 40, 60, 80 or 100 μL. Values are mean ± SEM of seven animals.

Animals were instrumented with an indwelling catheter into the lower lumbar spinal subarachnoid space by modifying our previously devised method [33] for the thoracic spinal cord in the rat. Briefly, animals were anesthetized in an induction chamber with an induction dose of 4% isoflurane (Abbot Laboratories, Abbot Park, IL, USA) using a Matrx VIP 3000 vaporizer (Midmark, Orchard Park, NY, USA). After induction, rats were placed on an operating table, and anesthesia was maintained with 2% isoflurane in 50% oxygen through a nose piece to carry out surgery. We removed the spinous process of L6 vertebra and drilled a hole through the cut portion of the lamina until the dura was exposed. A slit was made by traversing the surface of the dura with the tip of a 30-gauge needle, resulting in leakage of clear cerebrospinal fluid (CSF). A PE-10 catheter (Clay Adams, Parsippany, NJ, USA) was then inserted into the slit and advanced cephalically until 0.5 cm of the catheter was lodged in the subarachnoid space, placing its tip below the middle portion of L6 vertebra (Figure 2a). Animals were returned to the animal room for postoperative recovery in individual cages after they received sodium penicillin (1000 IU; YF Chemical Corporation, Taipei, Taiwan) given intramuscularly to prevent postsurgical infection, and were allowed free access to standard rat chow and water. Only animals that showed full recovery after 7 days were used in subsequent experiments.

### 2.5. Intrathecal Administration

During the recording sessions, the sealed end of the catheter was retrieved through a small skin incision under local anesthesia, and was first flushed with 10 μL of artificial CSF (aCSF) to ensure patency. This was followed by intrathecal administrations of either a contrast medium or bupivacaine into the spinal subarachnoid space, delivered at a rate of 10 μL/min by an infusion pump (CMA/102; CMA Microdialysis, Stockholm, Sweden).

### 2.6. Myelogram

Myelographic examination was performed in Group 1 animals under 1.5% isoflurane anesthesia on seven rats with the implanted subarachnoid catheter, using the Ultimax-IDREX-U180 digital X-ray system (Toshiba Medical Systems Corporation, Otawara, Japan). Four successive intrathecal administrations of a contrast medium (Opaque; GE Health, Cork, Ireland) were delivered at incremental volumes of 40, 60, 80 and 100 μL. Radiographs were taken in the prone position 2 min after each dosing, and the distribution of the contrast medium in the subarachnoid space was recorded.

### 2.7. General Preparation for Physiological Experiments

Animals in Groups 2, 3 and 4 similarly received an induction dose of 4% isoflurane, were placed on an operating table and were maintained under 2% isoflurane in 50% oxygen through a nose piece to carry out preparatory surgery. This included cannulation of the right femoral artery with a PE-20 catheter (Clay Adams, Parsippany, NJ, USA) to measure arterial pressure and isolation of the right common carotid artery for measurement of the blood flow. After surgery, animals were placed in a supine position on a platform with a heating pad and their rectal temperature was maintained at 37 ± 0.5 °C.

### 2.8. Measurement of Blood Pressure, Heart Rate and Spontaneous Baroreflexes

During the recording sessions, anesthesia was maintained at 1.5% isoflurane in 50% oxygen, delivered via a rat anesthesia mask. The recorded BP signals from the femoral artery were processed by an arterial BP analyzer (APR31a, Notocord, Croissy-Sur-Seine, France) to obtain systolic BP (SBP), HR and pulse interval (PI). SBP signals were simultaneously subjected to online and real-time spectral analysis (SPA10a, Notocord) to detect the power density of the low-frequency component (BLF; 0.25–0.8 Hz) of SBP spectrum. The power density of this spectral band has been demonstrated to be a valid experimental index for spontaneous baroreflex-mediated sympathetic vasomotor tone [34,35]. To evaluate the spontaneous cardiac vagal baroreflex, we employed a baroreflex sequence analyzer (BRS10a, Notocord, Croissy-Sur-Seine, France) to determine baroreflex sensitivity (BRS) based on online computerized scanning in search of reflex changes of PI in response to spontaneous sequences of consecutive increases or decreases in SBP [36]. We also calculated the baroreflex efficiency index (BEI), which reflects the number of times changes in HR are evoked in response to BP fluctuations over a time-window of 60 s. Concurrent temporal changes in SBP, MAP and HR were continuously recorded, alongside power density of the BLF band, BRS or BEI. To avoid potential bias created by nonstationary disturbances, only stationary segments of data from spectral analysis were used for statistical analysis.

### 2.9. Measurement of Carotid Blood Flow

We measured carotid blood flow (CBF) in Group 2 and 4 animals using a transit-time blood flowmeter (TS420, Transonic, Ithaca, NY, USA). The transonic flow probe (1.0 mm V-series; Transonic, Ithaca, NY, USA) was placed around the right common carotid artery to record the flow volume in mL/min. Anesthesia was maintained at 1.5% isoflurane during the recording session.

### 2.10. Measurement of Microvascular Perfusion, Tissue Oxygen Level and Temperature in the Cerebral Cortex

Because of the special positions for measuring probes, measurement of microvascular perfusion, tissue oxygen level and temperature in the cerebral cortex simultaneous with CBF determination is not feasible and has to be carried out in separate experiments (Group 3). A combined oxygen/temperature/blood flow probe designed for simultaneous and continuous measurement of tissue oxygen tension (PO_2_), blood flow and temperature (BF/OF/E; Oxford Optronix, Abingdon, UK) was stereotaxically positioned in an area of the cerebral cortex that exhibited severe infarction on occlusion of the middle cerebral artery [37]. Real-time microvascular red blood cell perfusion in tissue was processed by an OxyFlo monitor (Oxford Optronix). The laser Doppler signals from the tissue were recorded in blood perfusion units, which is a relative unit defined against a controlled motility standard. Instantaneous changes in local oxygen tension, compensated for fluctuations in tissue temperature, were processed by an OxyLite monitor (Oxford Optronix).

### 2.11. Sample Size Calculation

As previously described [38], we determined that the minimal number of animals of each of the three groups was seven based on the equation: *n* = 2σ^2^(Z_α/2_ + Z_1-β_)^2^/(μ1 − μ2)^2^, where the α level is 0.05; the pooled standard deviation (σ2) is 0.64; the normal deviate at 5% significance (Z_α/2_) is 1.96; the normal deviate at 80% statistical power (Z_1-β_) is 0.84; and the estimated difference between two means (μ1 − μ2) is 1.2.

### 2.12. Statistical Analyses

All values are expressed as means ± SEM. The averaged values calculated in each response phase (basal condition, Phase I, II or III) after intrathecal administration of bupivacaine, or four 40-min intervals after isoflurane anesthesia alone, were used for statistical analyses using SPSS version 22.0 (IBM Corp., Armonk, NY, USA). Two-way analysis of variance with repeated measures was first used to assess the group means, followed by the Dunnett (versus data obtained under basal conditions or the first 40 min) or Tukey (versus data obtained during Phase I) multiple-range test for post hoc assessment of individual means. *p* < 0.05 was taken to indicate statistical significance.

## 3. Results

### 3.1. Part 1. Patients

We present here three patients who developed cerebral infarction 4–8 h after surgery under spinal bupivacaine anesthesia. Postoperative examinations after the stroke incidents revealed that a common pathology shared by all three patients is stenosis or atheromatous lesions in carotid or middle cerebral artery.

Patient A had experienced two cerebral ischemic attacks, 12 years and 1 year prior to this admission. Postoperative transcranial ultrasound examination revealed high-grade stenosis in the right internal carotid artery and atheromatous lesions in the bilateral common carotid artery and left internal carotid artery. She developed right side weakness 5 h after discharge from the PACU. Diagnosis based on MRI was acute infarction of left fronto-parietal area with multiple vessel abnormality.

Patient B had no prior history of cerebrovascular incidence. He developed right side weakness 4 h after PACU discharge. Diagnosis based on postoperative MRI was small acute left corona radiate and basal ganglia infarcts. Carotid duplex study revealed atheromatosis in bilateral carotid artery bulb and left internal carotid artery.

Patient C had a history of over 50% stenosis in the left middle cerebral artery based on transcranial ultrasound examination, although no prior cerebrovascular episode was noted. He developed left side weakness 8 h after PACU discharge. Diagnosis based on postoperative MRI was acute infarction in the right pons.

### 3.2. Part 2. Animals

The postoperative examination after the stroke incidents that revealed a common pathology shared by patients who develop cerebral infarction after surgery under spinal bupivacaine anesthesia is stenosis or atheromatous lesions in carotid or middle cerebral artery prompted us to assess in a companion animal study whether intrathecal administration of bupivacaine compromises carotid and cerebral blood flow and whether baroreflex dysfunction plays a contributory role.

#### 3.2.1. Experimental Setup

Because of the special requirements for the experimental setup (Figure 1), male Sprague-Dawley rats that were instrumented with an indwelling catheter for intrathecal injection into the spinal subarachnoid space were randomly assigned to three groups (see relevant sections in the Materials and Methods for a detailed description). Group 1 animals received myelographic examination to establish the segmental distribution of intrathecally administered bupivacaine in the spinal cord. Group 2 and 3 were employed to determine the time-course alterations of MAP, HR, baroreflex-mediated sympathetic vasomotor tone, cardiac vagal baroreflex or BEI, simultaneous with CBF (Group 2) or tissue perfusion, oxygen and temperature in the cerebral cortex supplied by middle cerebral artery (Group 3), in response to intrathecal administration of bupivacaine. Group 4 (control group) animals were used to determine the time-course alterations hemodynamic parameters, baroreflex and CBF or tissue perfusion, oxygen or temperature in the cerebral cortex under 1.5% isoflurane anesthesia alone.

#### 3.2.2. Distribution of Contrast Medium in Spinal Subarachnoid Space after Intrathecal Administration

To set the stage for our animal study, we first established the segmental distribution of intrathecally administered bupivacaine to induce spinal anesthesia in rats. For this purpose, we employed myelographic examination to determine the relationship between the volume of administration and extent of dispersion in the spinal cord using a contrast medium as the surrogate (Figure 2b,c). Taking into consideration that the segments of spinal cord always lie higher than the corresponding vertebrae [39], injection of 40 μL of contrast medium via the tip of our catheter lodged at the subarachnoid space below L6 vertebra (Figure 2a) manifested an enhanced roentgenological image at the S2 spinal cord (Figure 2(b2,c)) as compared with the pre-enhanced image (Figure 2(b1)). Increasing the volume of administered contrast medium to 60, 80 or 100 μL extended the enhanced image to the L1 (Figure 2(b3,c)), T10 (Figure 2(b4,c)) or T2 (Figure 2(b5,c)) spinal cord. Based on those observations, we chose 80 μL as the volume of bupivacaine injection in subsequent physiological experiments because its distribution in the spinal cord closely mimics the targeted segmental levels of spinal anesthesia in our patients.

#### 3.2.3. Common Response Pattern of Carotid or Cerebral Blood Flow and Baroreflex Functionality to Spinal Bupivacaine

**Figure 3:**
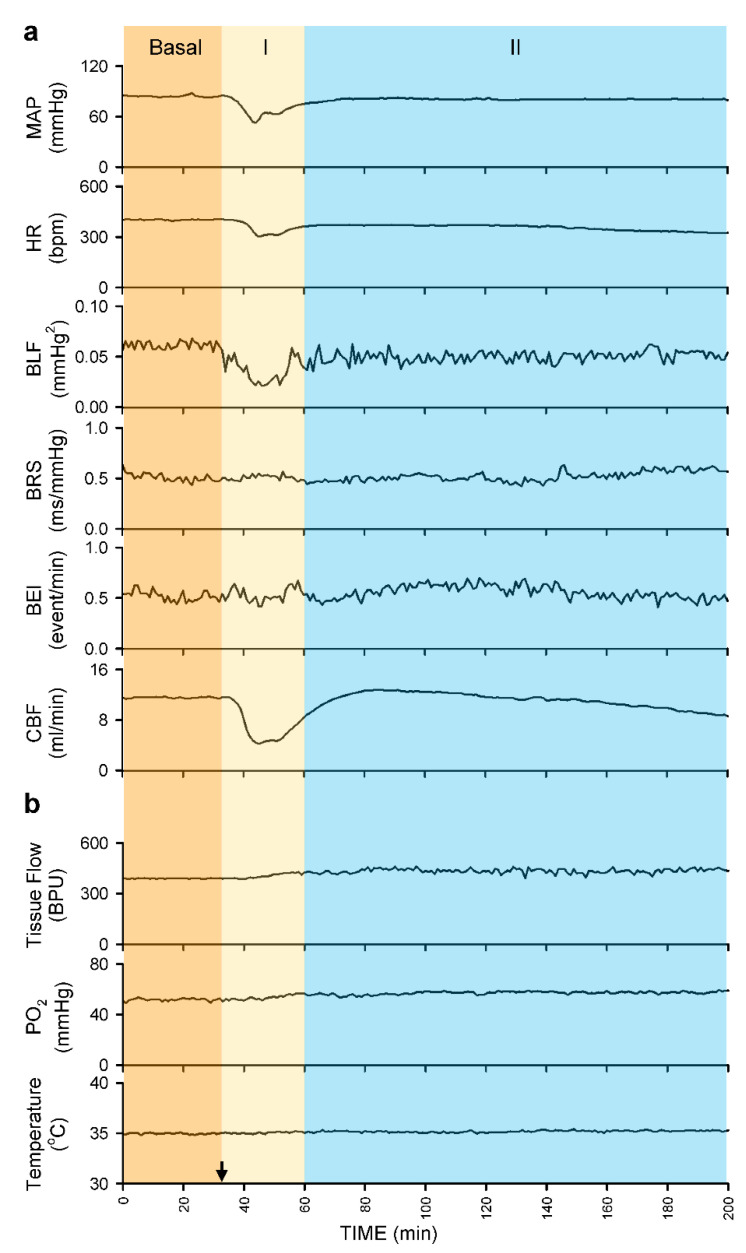
Illustrative examples of real-time and online recording of common phasic changes in mean arterial pressure (MAP), heart rate (HR), power density of the low-frequency component in systolic blood pressure spectrum (BLF), baroreflex sensitivity (BRS) or baroreflex effectiveness index (BEI), simultaneous with carotid blood flow (CBF) in Group 2 animals (**a**); or concurrent with tissue perfusion (Tissue Flow), tissue oxygen tension (PO_2_) or tissue temperature in the cerebral cortex in Group 3 animals (**b**) on intrathecal administration of 80 μL of bupivacaine (at arrow).

**Figure 4:**
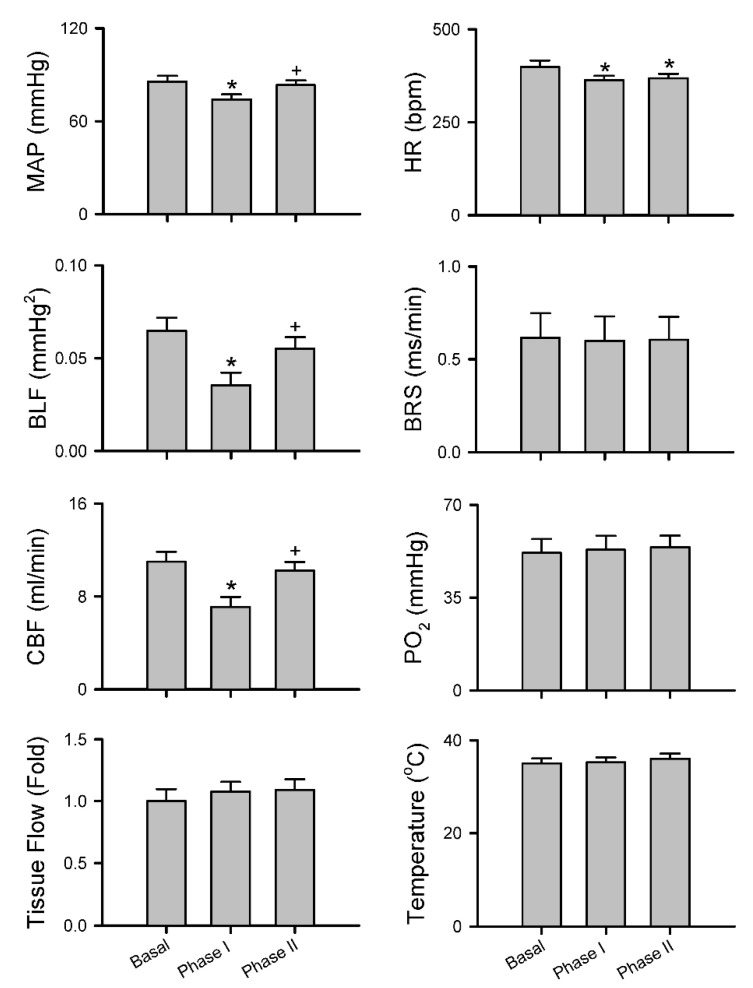
Common response patterns of MAP, HR, BLF, BRS and CBF or tissue perfusion, PO_2_ or temperature in the cerebral cortex to intrathecal administration of 80 μL of bupivacaine. Note that values for MAP, HR, BLF and BRS are mean ± SEM of 33 animals (17 from Group 2 plus 16 from Group 3); values for CBF are mean ± SEM of 17 animals from Group 2; and values for tissue perfusion PO_2_ or temperature in the cerebral cortex are mean ± SEM of 16 animals from Group 3. * *p* < 0.05 versus data obtained under basal conditions in the post hoc Dunnett multiple-range analysis; ^+^ *p* < 0.05 versus data obtained during Phase I in the post hoc Tukey multiple-range analysis.

**Figure 5:**
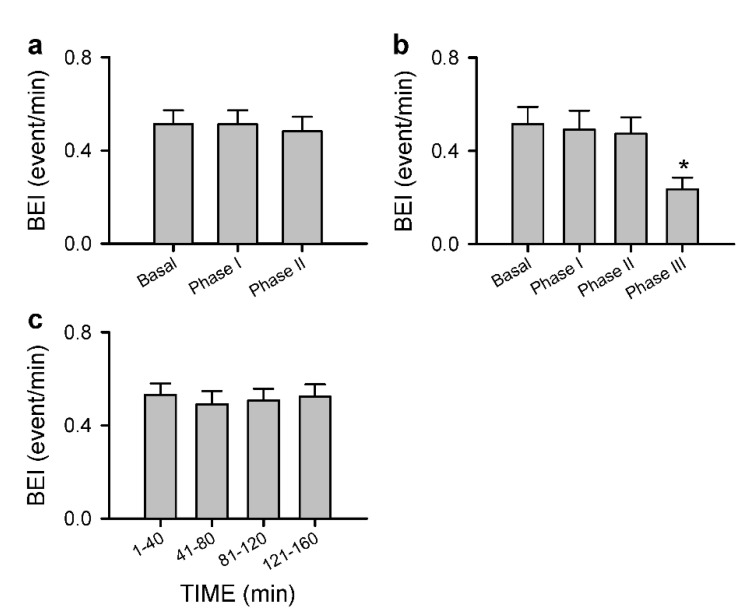
Common (**a**) and anomalous (**b**) response patterns of BEI to intrathecal administration of 80 μL of bupivacaine. Values for (**a**) are mean ± SEM from 33 animals (17 from Group 2 plus 16 from Group 3); and values for (**b**) are mean ± SEM of 15 animals (7 from Group 2 plus 8 from Group 3). * *p* < 0.05 versus data obtained under basal conditions in the post hoc Dunnett multiple-range analysis. (**c**) Insignificant changes of BEI under isoflurane alone without spinal anesthesia. Values are mean ± SEM of 14 animals from Group 4. No significance among all groups (*p* > 0.05).

**Figure 6:**
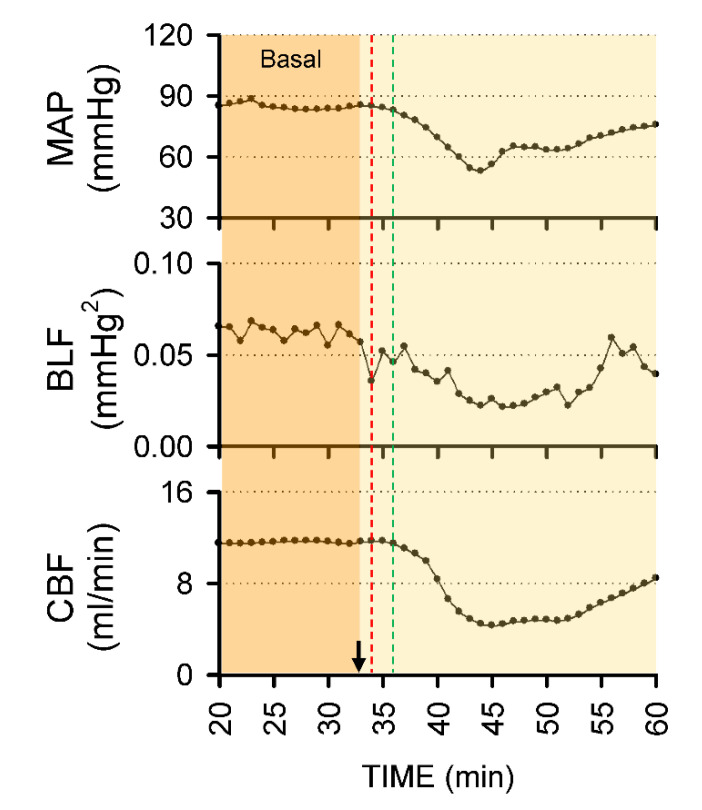
Zoomed-in view of 40 min of real-time recording from Figure 2a of Phase I changes in MAP, BLF and CBF on intrathecal administration of 80 μL of bupivacaine (at arrow). Note that the red and green dotted lines denote time points at which reduction of BLF power and MAP or CBF commenced.

Intrathecal administration of 80 μL of bupivacaine resulted in two distinct phases of temporal changes in cardiovascular events, CBF and baroreflex functionality in 17 of 24 (70.8%) animals in Group 2 studied (Figure 3, Figure 4 and Figure 5). Phase I, which endured 20 min, manifested a significant reduction in MAP and HR, accompanied by a significant decrease in CBF and power density of BLF component, an experimental index for baroreflex-mediated sympathetic vasomotor tone, without significant alterations in BRS, an experimental index for cardiac vagal baroreflex (Figure 3a and Figure 4). Closer examination of expanding the time-scale of Figure 3a revealed that the reduction in BLF power actually took place before the reduction in MAP and CBF (Figure 6). Phase II, which exhibited a return of MAP, CBF or power density of BLF component to the basal level amid a progressive decrease in HR, lasted for the remainder of the 160 min observation period (Figure 3a and Figure 4). Of note is that BEI presented no significant alterations during Phases I and II as compared to its basal level (Figure 5a).

In a separate series of experiments (Figure 3b, Figure 4 and Figure 5a), intrathecal administration of 80 μL of bupivacaine in 16 of 24 (66.7%) animals in Group 3 studied resulted in phasic changes in MAP, HR, BEI and BLF power or BRS that were comparable to those depicted above. Of interests is that local blood flow, tissue oxygen tension or temperature in the cerebral cortex remained similar to their basal levels during both Phases I and II.

#### 3.2.4. Anomalous Response Pattern of Carotid or Cerebral Blood Flow and Baroreflex Functionality to Spinal Bupivacaine

**Figure 7:**
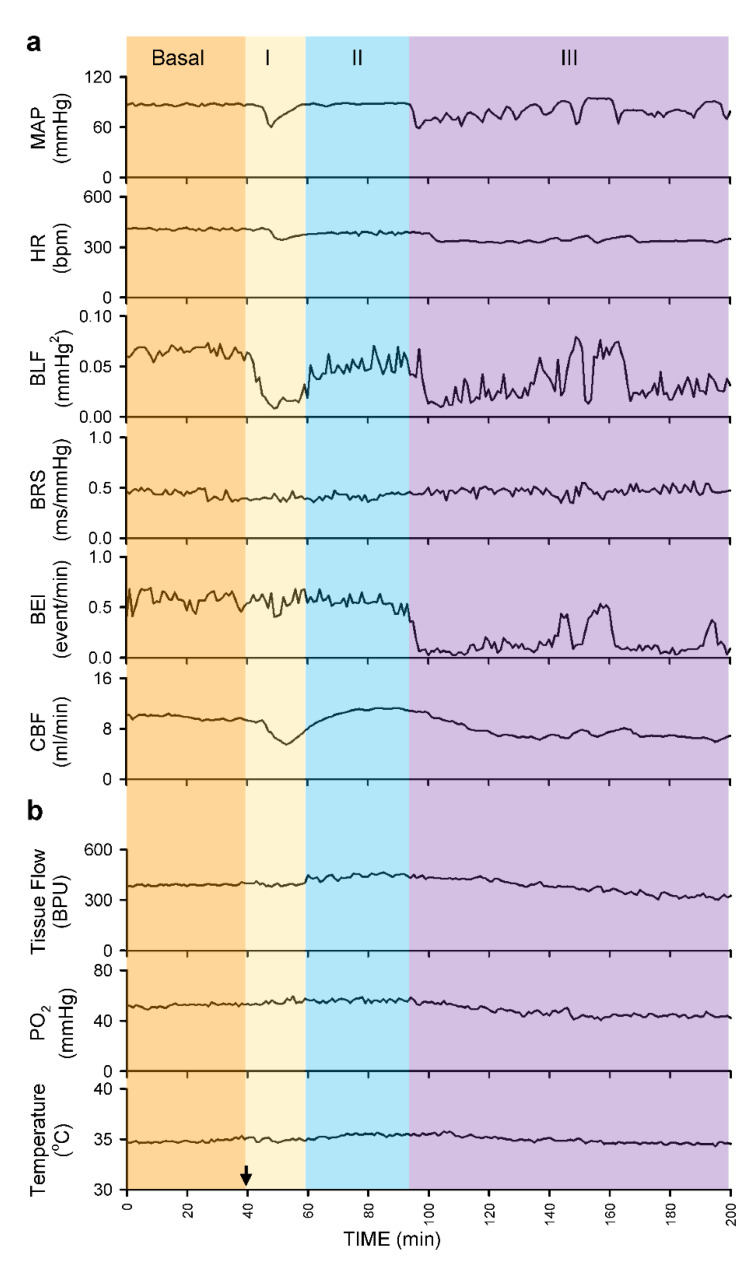
Illustrative examples of real-time and online recording of anomalous phasic changes in MAP, HR, BLF, BRS or BEI, simultaneous with CBF in Group 2 animals (**a**); or concurrent with tissue perfusion, PO_2_ or temperature in the cerebral cortex in Group 3 animals (**b**) with intrathecal administration of 80 μL of bupivacaine (at arrow).

**Figure 8:**
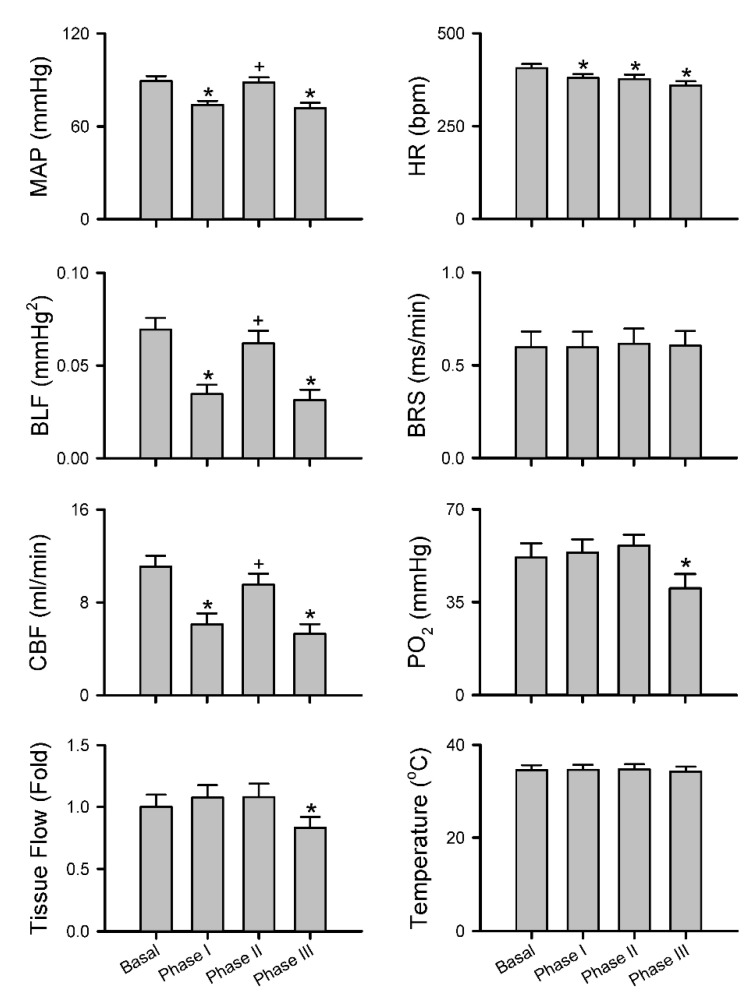
Anomalous response patterns of MAP, HR, BLF, BRS and CBF or tissue perfusion, PO_2_ or temperature in the cerebral cortex to intrathecal administration of 80 μL of bupivacaine. Note that values for MAP, HR, BLF and BRS are mean ± SEM of 15 animals (7 from Group 2 plus 8 from Group 3); values for CBF are mean ± SEM of 7 animals from Group 2; and values for tissue perfusion, PO_2_ or temperature in the cerebral cortex are mean ± SEM of 8 animals from Group 3. * *p* < 0.05 versus data obtained under basal conditions in the post hoc Dunnett multiple-range analysis; + *p* < 0.05 versus data obtained during Phase I in the post hoc Tukey multiple-range analysis.

We also encountered, in 15 of 48 (31.3%) animals studied (7 from Group 2 plus 8 from Group 3), an unanticipated anomalous response pattern to intrathecal administration of 80 μL of bupivacaine. Whereas the biphasic response patterns of MAP, HR, BLF power, BRS or BEI in those rats were comparable to those depicted in Figure 3b, Figure 4 and Figure 5a, Phase II gave way to Phase III after sustaining for less than 40 min (Figure 5b, Figure 7a and Figure 8). Phase III, which persisted until the end of our 160 min observation period, was characterized by a significant secondary hypotension and reduction of baroreflex-mediated sympathetic vasomotor tone, alongside a continuous decline in HR, sustained cardiac vagal baroreflex and significant reduction in BEI. Of note is that the waxing and waning alterations in MAP, BLF power and BEI were manifested in a correlated manner. CBF also underwent a secondary decrease (Figure 7a and Figure 8) in 7 of 24 (29.2%) animals in Group 2. Likewise, 8 out of 24 (33.3%) Group 3 rats exhibited a significant decrease in local blood flow or tissue oxygen tension in the cerebral cortex (Figure 7b and Figure 8) during Phase III.

#### 3.2.5. Insignificant Changes in Carotid or Cerebral Blood Flow and Baroreflex Functionality under Isoflurane Anesthesia

**Figure 9:**
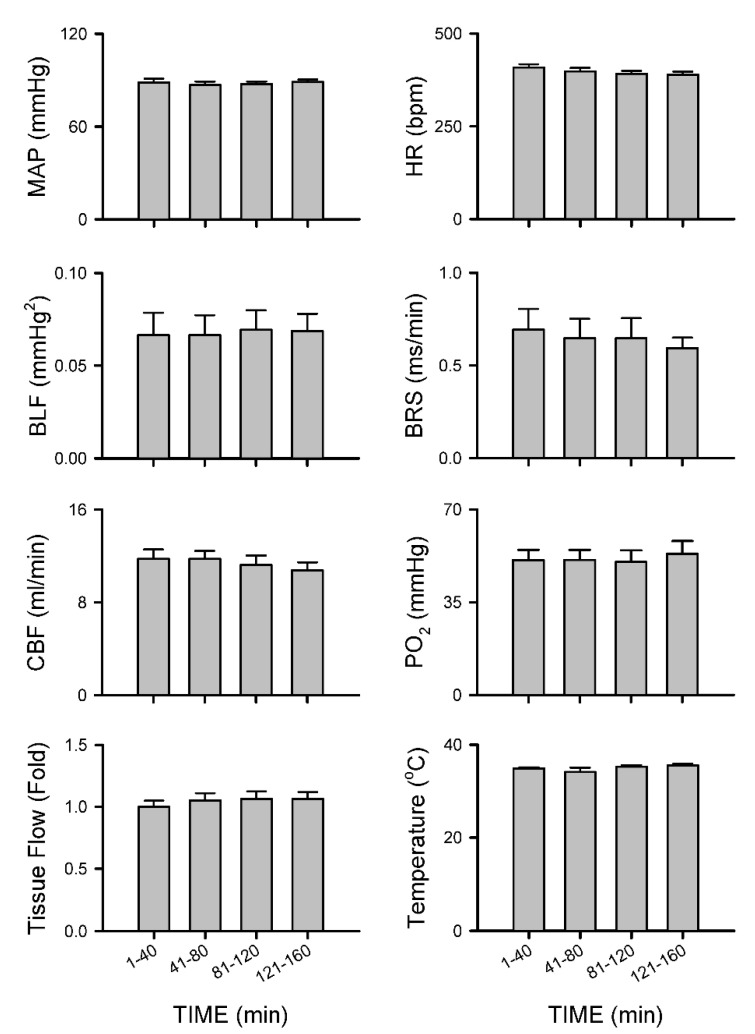
Insignificant changes of MAP, HR, BLF, BRS and CBF or tissue perfusion, PO_2_ or temperature in the cerebral cortex under 1.5% isoflurane anesthesia alone. Note values for MAP, HR, BLF and BRS are mean ± SEM of 14 animals (7 from Group 4 CBF experiments plus 7 from Group 4 tissue perfusion experiments); values for CBF are mean ± SEM of 7 animals; and values for tissue perfusion, PO_2_ or temperature in the cerebral cortex are mean ± SEM of 7 animals. No significance among all groups (*p* > 0.05).

Group 4 rats that were subjected to 1.5% isoflurane anesthesia alone without additional intrathecal administration of bupivacaine exhibited insignificant changes in MAP, HR, BLF power, BRS and CBF and tissue perfusion, tissue oxygen tension or temperature in the cerebral cortex (Figure 9) or BEI (Figure 5c) over our 160 min observation period.

## 4. Discussion

Spinal anesthesia is generally accepted as an effective and safe technique by anesthesiologists and surgeons because the anesthetic effect can be confined to lower thoracic or lumbar dermatomes of the body and interference to cardiovascular functions is usually benign and readily correctable. With the exception of infants [25] or elderly [24], it is also generally conceived that spinal anesthesia would not have discernible effect on cerebral blood supply. Based on complementary observations from patients and animals, our study revealed that those notions may have to be modified because carotid and cerebral blood flow can, indeed, be compromised after spinal anesthesia with bupivacaine and baroreflex dysfunction plays a contributory role.

Steel observed in 1925 [40] that “A fall of blood pressure accompanies each spinal anesthesia… Its low point is usually ten minutes after the injection, and most fatalities have occurred at that time. After fifteen minutes, one is working away from the danger point, not toward it as in other general anesthetics.” Amazingly, this almost century-old observation is still valid today, and results from our animal study offer a plausible underlying physiological scenario. We demonstrated that on intrathecal administration of 80 μL of bupivacaine that reached T10 spinal cord, there was indeed a transient decrease of MAP (Phase I) in all rats from Groups 2 and 3. As a consequence of this hypotension, CBF underwent a transitory reduction. On the other hand, cerebral blood flow and tissue oxygen tension remained stable, indicating that cerebral autoregulation was operational. Baroreflex-mediated sympathetic vasomotor tone and cardiac vagal baroreflex are, respectively, compensatory reflex changes in vascular tone and HR in response to alteration of BP. Our results, nonetheless, indicated that the two arms of baroreflex play differential roles in response to spinal anesthesia. The essentially unaltered BRS and BEI during Phase I implied that, as reported previously [24], cardiac vagal baroreflex in low spinal anesthesia is not responsible for the induced bradycardia. Instead, the transient decrease in HR is possibly related to the reverse Bainbridge reflex [18] because of the dilatation of veins by the bupivacaine-induced preganglionic sympathetic paralysis [11]. On the other hand, as indicated by the reduction of BLF power that preceded the decrease in MAP (Figure 6), hypotension may be accounted for by the temporary curtailment of the baroreflex-mediated vasomotor tone to exert its tonic actions on systemic vascular resistance because of the elicited sympathetic block [13,14,15,16]. During Phase II, “…working away from the danger point” takes place on resumption of baroreflex-mediated vasomotor tone, leading to a significant return of MAP and CBF towards baseline, amid sustained bradycardia.

Postoperative stroke is rare, but its consequence is devastating. It is generally considered that postoperative stroke is multifactorial, and intraoperative hypotension may aggravate the pathological process by increasing the infarction size [26,27]. Furthermore, a decrease of MAP by more than 30% from baseline is significantly associated with the occurrence of a postoperative stroke [41], and watershed infarct is associated with hypotension following spinal anesthesia [42]. By postoperative MRI examination, we confirmed that watershed infarct is absent in the three patients. On the other hand, we are aware that the secondary decline in CBF during Phase III in Group 2 animals is reminiscent of the potentially impeded blood flow in their carotid or middle cerebral artery because of stenosis or atheromatous lesions in these vessels. Judging from the significant and correlative waxing and waning alterations in MAP, BLF power and BEI, it is conceivable that the retarded effectiveness of baroreflex-mediated sympathetic vasomotor tone during Phase III underpins the secondary hypotension that leads to the reduction in CBF. It follows that, coupled with dysfunctional cerebral autoregulation, the significant decrease in local tissue blood flow or tissue oxygen tension in the cerebral cortex supplied by the middle cerebral artery offers a plausible modus operandi, albeit differences in time-windows (minutes versus hours), for the unanticipated cerebral infarction in our three reported cases. This notion is in line with the observation [43] that dynamic autoregulation is impaired in a subgroup of patients with carotid artery stenosis, who are at risk from subsequent stroke, as demonstrated by their inability to maintain middle cerebral artery blood flow in response to a rapid reduction of BP. Because of the maintained cardiac vagal baroreflex, it is also likely that the secondary decrease in HR is sustained by a reverse Bainbridge reflex [18].

We are cognizant that our study design does not allow us to offer a mechanistic explanation for the transition between the phases in Groups 2 and 3 animals. Four pieces of information from the physiological and anatomical literature afford some speculations. First, it is well known that sympathetic vasomotor tone is generated by a tonic excitatory action from the rostral ventrolateral medulla onto the intermediolateral nucleus, where 85% of the sympathetic preganglionic neurons (SPNs) originate, subjected to modulation by inputs from the baroreceptor afferents via the nucleus tractus solitarii [44]. Second, superimposed on this well-known phenomenon, we reported previously [34] that both nitric oxide synthase I and II (NOS I and NOS II) are present in the thoracic spinal cord and are tonically active. In particular, the endogenous NO generated by NOS I-containing SPNs exerts a tonic excitatory action on the vasomotor tone mediated by norepinephrine released from the adrenal medulla and sympathetic nerve terminals. On the other hand, NO, derived from NOS II-containing fibers that originate from the rostral ventrolateral medulla and impinge on SPNs, exerts a tonic inhibitory action on the sympathetic outflow that primarily targets the blood vessels. Third, the spinal origin of SPNs to the celiac ganglion in the rat, which regulates splanchnic circulation, is T4 to T13; that to the adrenal gland is T4 to T12 [45]. Interestingly, more than 60% of the SPNs that project to the celiac ganglion exhibit NOS I-immunoreactivity [46]. Fourth, sympathetic input to the splanchnic vasculature is not critical for normal functions of the splanchnic organs, but instead mainly participates in the overall regulation of the circulation [47]. Speculatively, the initial brunt of intrathecal administration of bupivacaine is paralysis of the NOS I-containing SPNs located below T10, resulting in the acute withdrawal of the tonic excitatory action on the splanchnic vasculature during Phase I. During Phase II, baroreflex-mediated vasomotor tone triggered by hypotension utilizes the intact NOS I-containing SPNs located above T10 as its output component via their innervation of the celiac ganglion and adrenal gland. The inadvertent shift of dominance towards NOS II activity that exerts an inhibitory action on SPNs may account for the events observed during Phase III. The correlative waxing and waning alterations in MAP, BLF power and BEI may in effect reflect the result of reciprocate balance between NOS I and NOS II activity. These speculations, however, are subject to further validation.

One limitation of this study is that our results do not conclusively show that spinal anesthesia is the sole reason for the secondary drop in cerebral blood flow in one-third of the animals. Isoflurane exposure was exactly the same for all animals in Groups 2 and 3 regardless of whether rats exhibited a decrease CBF or tissue perfusion in the cerebral cortex on intrathecal administration of bupivacaine. Together with the lack of insignificant changes in these two flow parameters in our control animals that received isoflurane alone, our results do implicate a role for spinal anesthesia in the secondary drop in carotid or cerebral blood flow. We also wish to point out that the goal of our study was to clarify whether spinal anesthesia with bupivacaine compromises carotid and cerebral blood flow in healthy rats. It was never our intention to study whether stenosis or atheromatous lesions in the carotid or middle cerebral artery, well-known risk factors for cerebral ischemic attack, will affect spinal anesthesia.

## 5. Conclusions

Based on complementary observations from patients and animals, we demonstrated that carotid and cerebral blood flow can, indeed, be compromised after spinal anesthesia with bupivacaine, and impaired baroreflex-mediated sympathetic vasomotor tone, which leads to hypotension, may play a contributory role. Spinal anesthesia is generally accepted to be a safe procedure; however, while postoperative stroke is rare, its consequence is devastating. Results from this study, therefore, post a cautionary note to this widely used anesthetic practice.

## Data Availability

The data presented in this study are available on reasonable request from the corresponding author.

## References

[1] Wulf H.F.W. (1998). The Centennial of Spinal Anesthesia. Anesthesiology.

[2] Konrad C., Schupfer G., Wietlisbach M., Gerber H. (1998). Learning manual skills in anesthesiology: Is there a recommended number of cases for anesthetic procedures?. Anesth. Analg..

[3] Kopacz D.J., Neal J.M., Pollock J.E. (1996). The regional anesthesia “learning curve”. What is the minimum number of epidural and spinal blocks to reach consistency?. Reg. Anesth..

[4] Memtsoudis S.G., Sun X., Chiu Y.-L., Stundner O., Liu S.S., Banerjee S., Mazumdar M., Sharrock N.E. (2013). Perioperative Comparative Effectiveness of Anesthetic Technique in Orthopedic Patients. Anesthesiology.

[5] Pugely A., Martin C.T., Gao Y., Mendoza-Lattes S., Callaghan J.J. (2013). Differences in Short-Term Complications Between Spinal and General Anesthesia for Primary Total Knee Arthroplasty. J. Bone Jt. Surg. Am. Vol..

[6] Van Waesberghe J., Stevanovic A., Rossaint R., Coburn M. (2017). General vs. neuraxial anaesthesia in hip fracture patients: A systematic review and meta-analysis. BMC Anesthesiol..

[7] Arndt J.O., Bomer W., Krauth J., Marquardt B. (1998). Incidence and time course of cardiovascular side effects during spinal anesthesia after prophylactic administration of intravenous fluids or vasoconstrictors. Anesth. Analg..

[8] Carpenter R.L., Caplan R.A., Brown D.L., Stephenson C., Wu R. (1992). Incidence and Risk Factors for Side Effects of Spinal Anesthesia. Anesthesiology.

[9] Gentili M., Huu P.C., Enel D., Hollande J., Bonnet F. (1998). Sedation depends on the level of sensory block induced by spinal anaesthesia. Br. J. Anaesth..

[10] Inagaki Y., Mashimo T., Kuzukawa A., Tsuda Y., Yoshiya I. (1994). Epidural lidocaine delays arousal from isoflurane anes-thesia. Anesth. Analg..

[11] Greene N.M. (1981). Preganglionic Sympathetic Blockade in Man: A Study of Spinal Anesthesia. Acta Anaesthesiol. Scand..

[12] Sancetta S.M., Lynn R.B., Simeone F.A., Scott R.W., Heckman G., Janouskovec H. (1952). Studies of Hemodynamic Changes in Humans Following Induction of Low and High Spinal Anesthesia. Circulation.

[13] Brooker R.F., Butterworth J.F., Kitzman D.W., Berman J.M., Kashtan H.I., McKinley A.C. (1997). Treatment of hypo-tension after hyperbaric tetracaine spinal anesthesia. A randomized, double-blind, cross-over comparison of phe-nylephrine and epinephrine. Anesthesiology.

[14] Butterworth J. (1998). Physiology of spinal anesthesia: What are the implications for management?. Reg. Anesth. Pain Med..

[15] Critchley L.A., Conway F. (1996). Hypotension during subarachnoid anaesthesia: Haemodynamic effects of colloid and me-taraminol. Br. J. Anaesth..

[16] Rooke G.A., Freund P.R., Jacobson A.F. (1997). Hemodynamic response and change in organ blood volume during spinal anesthesia in elderly men with cardiac disease. Anesth. Analg..

[17] Critchley L.A., Chan S., Tam Y.H. (1998). Spectral analysis of sudden bradycardia during intrathecal meperidine anesthesia. Reg. Anesth. Pain Med..

[18] Crystal G.J., Salem M.R. (2012). The Bainbridge and the "reverse" Bainbridge reflexes: History, physiology, and clinical rele-vance. Anesth. Analg..

[19] Mather L.E., Chang D.H. (2001). Cardiotoxicity with modern local anaesthetics: Is there a safer choice?. Drugs.

[20] Cowley A.W., Liard J.F., Guyton A.C. (1973). Role of baroreceptor reflex in daily control of arterial blood pressure and other variables in dogs. Circ. Res..

[21] Thrasher T.N., Burattini R., Borgdorff P., Westerhof N. (2004). Baroreceptors and the long-term control of blood pressure. Exp. Physiol..

[22] McHenry L.C., West J.W., Cooper E.S., Goldberg H.I., Jaffe M.E. (1974). Cerebral autoregulation in man. Stroke.

[23] Gratadour P., Viale J.P., Parlow J., Sagnard P., Counioux H., Bagou G., Annat G., Hughson R., Quintin L. (1997). Sym-pathovagal effects of spinal anesthesia assessed by the spontaneous cardiac baroreflex. Anesthesiology.

[24] Minville V., Asehnoune K., Salau S., Bourdet B., Tissot B., Lubrano V., Fourcade O. (2009). The Effects of Spinal Anesthesia on Cerebral Blood Flow in the Very Elderly. Anesth. Analg..

[25] Bonnet M.-P., Larousse E., Asehnoune K., Benhamou D. (2004). Spinal Anesthesia with Bupivacaine Decreases Cerebral Blood Flow in Former Preterm Infants. Anesth. Analg..

[26] Kam P.C.A., Calcroft R.M. (1997). Peri-operative stroke in general surgical patients. Anaesthesia.

[27] Parikh S., Cohen J.R. (1993). Perioperative stroke after general surgical procedures. N. Y. State J. Med..

[28] Selim M. (2007). Perioperative stroke. N. Engl. J. Med..

[29] Szeder V., Torbey M.T. (2008). Prevention and Treatment of Perioperative Stroke. Neurologist.

[30] Knapp R.B., Topkins M.J., Artusio J.F. (1962). The Cerebrovascular Accident and Coronary Occlusion in Anesthesia. JAMA.

[31] Larsen S.F., Zaric D., Boysen G. (1988). Postoperative cerebrovascular accidents in general surgery. Acta Anaesthesiol. Scand..

[32] Thoennissen J., Herkner H., Lang W., Domanovits H., Laggner A.N., Müllner M. (2001). Does bed rest after cervical or lumbar puncture prevent headache? A systematic review and meta-analysis. Can. Med. Assoc. J..

[33] Poon Y.Y., Chang A.Y.W., Ko S.F., Chan S.H.H. (2005). An Improved Procedure for Catheterization of the Thoracic Spinal Subarachnoid Space in the Rat. Anesth. Analg..

[34] Poon Y.-Y., Tsai C.-Y., Cheng C.-D., Chang A.Y.W., Chan S.H.H. (2016). Endogenous nitric oxide derived from NOS I or II in thoracic spinal cord exerts opposing tonic modulation on sympathetic vasomotor tone via disparate mechanisms in anesthetized rats. Am. J. Physiol. Circ. Physiol..

[35] Li P.-L., Chao Y.-M., Chan S.H.H., Chan J.Y.H. (2001). Potentiation of Baroreceptor Reflex Response by Heat Shock Protein 70 in Nucleus Tractus Solitarii Confers Cardiovascular Protection During Heatstroke. Circulation.

[36] Laude D., Baudrie V., Elghozi J.-L. (2008). Applicability of recent methods used to estimate spontaneous baroreflex sensitivity to resting mice. Am. J. Physiol. Integr. Comp. Physiol..

[37] Mukda S., Tsai C.-Y., Leu S., Yang J.-L., Chan S.H.H. (2019). Pinin protects astrocytes from cell death after acute ischemic stroke via maintenance of mitochondrial anti-apoptotic and bioenergetics functions. J. Biomed. Sci..

[38] Poon Y.-Y., Tsai C.-Y., Huang Y., Wu J.C.C., Chan S.H.H., Chan J.Y.H. (2021). Disproportional cardiovascular depressive effects of isoflurane: Serendipitous findings from a comprehensive re-visit in mice. Lab Anim..

[39] Gelderd J.B., Chopin S.F. (1977). The vertebral level of origin of spinal nerves in the rat. Anat. Rec. Adv. Integr. Anat. Evol. Biol..

[40] Steel W.A. (1925). Blood Pressure Maintenance in Spinal Anesthesia. J. Am. Med. Assoc..

[41] Bijker J.B., Persoon S., Peelen L.M., Moons K.G., Kalkman C.J., Kappelle L.J., van Klei W.A. (2012). Intraoperative hypo-tension and perioperative ischemic stroke after general surgery: A nested case-control study. Anesthesiology.

[42] Abkur T.M., Mohamed M.B., Peters C. (2014). Multiple territory watershed infarcts following spinal anaesthesia. BMJ Case Rep..

[43] White R.P., Markus H.S. (1997). Impaired dynamic cerebral autoregulation in carotid artery stenosis. Stroke.

[44] Guyenet P.G., Stornetta R.L., Bochorishvili G., Depuy S.D., Burke P.G., Abbott S.B. (2013). C1 neurons: The body’s EMTs. Am. J. Physiol. Regul. Integr. Comp. Physiol..

[45] Strack A., Sawyer W., Marubio L., Loewy A. (1988). Spinal origin of sympathetic preganglionic neurons in the rat. Brain Res..

[46] Domoto T., Teramoto M., Tanigawa K., Tamura K., Yasui Y. (1995). Origins of nerve fibers containing nitric oxide synthase in the rat celiac-superior mesenteric ganglion. Cell Tissue Res..

[47] Fink G.D., Osborn J.W. (2012). The Splanchnic Circulation.

